# Diabetes risk reduction diet and the risk of breast cancer

**DOI:** 10.1097/CEJ.0000000000000709

**Published:** 2021-08-16

**Authors:** Federica Turati, Francesca Bravi, Marta Rossi, Diego Serraino, Veronica Mattioli, Livia Augustin, Anna Crispo, Attilio Giacosa, Eva Negri, Carlo La Vecchia

**Affiliations:** a Unit of Medical Statistics and Biometry, Fondazione IRCCS Istituto Nazionale Dei Tumori Di Milano; b Department of Clinical Sciences and Community Health, Università degli Studi di Milano, Milan; c Unit of Cancer Epidemiology, CRO Aviano National Cancer Institute, IRCCS, Aviano; d Epidemiology and Biostatistics Unit, Istituto Nazionale dei Tumori – IRCCS “Fondazione G. Pascale”, Naples; e Department of Gastroenterology and Clinical Nutrition, Policlinico di Monza, Monza; f Department of Humanities, Pegaso Online University, Naples, Italy

**Keywords:** breast neoplasms, case-control studies, diabetes mellitus, diet, epidemiology, primary prevention, risk factors

## Abstract

**Objective:**

Diabetes and insulin levels may increase the risk of postmenopausal breast cancer. In the present investigation, we aimed at evaluating whether adherence to a diabetes risk reduction diet (DRRD) lowers the risk of breast cancer.

**Methods:**

We used data from an Italian, multicentric case-control study (1991–1994) including 2569 incident histologically-confirmed breast cancer cases and 2588 hospital controls. A food frequency questionnaire collected subjects’ usual diet. We derived a DRRD score on the basis of eight items: intake of cereal fiber, total fruit, coffee, polyunsaturated to saturated fats ratio and nuts (higher scores for higher intakes), and dietary glycemic index, red/processed meat and sugar-sweetened beverages/fruit juices (higher scores for lower intakes). The score theoretically ranged 8–37, with higher values indicating greater DRRD adherence. Odds ratios (ORs) of breast cancer according to the DRRD score were estimated using multiple logistic regression models.

**Results:**

The DRRD score was inversely related to the risk of breast cancer. The ORs were 0.93 [95% confidence interval (CI), 0.89–0.98] for a three-point score increment and 0.76 (95% CI, 0.64–0.89) for the highest versus the lowest quartile (*P* for trend 0.001). Inverse associations were observed in subgroups of covariates.

**Conclusions:**

Higher DRRD adherence may decrease the risk of breast cancer.

## Introduction

Type II diabetes has been associated to a modest increased risk of postmenopausal breast cancer in several studies. Diabetic subjects have a 10–30% excess risk of postmenopausal breast cancer, likely explained by residual confounding by adiposity (La Vecchia *et al.*, 2011; Boyle *et al.*, 2012), a predisposing factor of both type II diabetes (Carey *et al.*, 1997) and postmenopausal breast cancer (Chan *et al.*, 2019; van den Brandt *et al.*, 2021). In premenopause, diabetes is not associated with breast cancer (Boyle *et al.*, 2012) and high BMI decreases the risk (van den Brandt *et al.*, 2021). Studies linking hallmark features of type II diabetes such as hyperglycemia, hyperinsulinemia and insulin resistance with breast cancer yielded mixed findings, suggesting direct as well as null associations (Hernandez *et al.*, 2014). Higher plasma levels of insulin-like growth factor-1 (IGF-1) may increase the risk of breast cancer, but, again, the evidence is not clear-cut (Schernhammer *et al.*, 2006; Key *et al*., 2010).

Insulin increases sex hormones and decreases sex hormone-binding globulin, which results in increased plasma-free steroid hormones concentrations, free estrogens in particular (Wolf *et al.*, 2005). High levels of endogenous sex hormones are associated with an excess risk of postmenopausal breast cancer (Key *et al.*, 2002). In addition, hyperinsulinemia secondary to insulin resistance may stimulate cellular signaling pathways with a role in tumorigenesis, including the AKT and extracellular-signal-regulated-kinase (ERK) pathways (Wolf *et al.*, 2005), and may increase IGF-1 expression, which is involved in the etiology and progression of cancer (Gallagher and LeRoith, 2010). A role of hyperglycemia and inflammatory cytokines has also been suggested (Giovannucci *et al.*, 2010).

Selected aspects of diet may have a certain role on breast cancer. Some specific dietary factors have been related to the disease, including alcohol and red and processed meat (Inoue-Choi *et al.*, 2016; Farvid *et al.*, 2018) among the unfavorable factors, and fruit and nonstarchy vegetables (Farvid *et al.*, 2016; Farvid *et al.*, 2019), dietary fiber (Farvid *et al.*, 2020) and carotenoids (Eliassen *et al.*, 2012) among the favorable ones; however, except for alcohol (Bagnardi *et al.*, 2015), evidence remains controversial. In addition, healthy dietary patterns considering simultaneously multiple aspects of diet, including the Mediterranean diet (Buckland *et al.*, 2013; Turati *et al.*, 2018) and a diet compliant with the nutritional recommendations from the World Cancer Research Fund/American Institute for Cancer Research (WCRF/AICR) (Turati *et al.*, 2020), have been associated to a reduced risk of breast cancer (Xiao *et al.*, 2019).

A dietary pattern developed for diabetes risk reduction [diabetes risk reduction diet (DRRD)] (Rhee *et al.*, 2015) characterized by high intakes of cereal fiber, coffee, fruit and nuts, a high ratio of polyunsaturated to saturated fats, and low dietary glycemic index (GI), low intakes of red/processed meat, sugar-sweetened beverages/fruit juices and trans fats showed a modest inverse association with the risk of breast cancer in a pooled analysis of two large US cohorts (Kang *et al.*, 2020). Further data are needed to clarify the issue.

In the current investigation, we evaluated whether a score measuring adherence to the DRRD lowers the risk of breast cancer using data from a large, multicentric case-control study conducted in a Mediterranean country.

## Methods

We used data from a multicentric case-control study on breast cancer conducted from June 1991 to April 1994 in six Italian areas: the provinces of Pordenone and Gorizia, the greater Milan area, the urban area of Genoa, the province of Forli, the province of Latina, and the urban area of Naples (Franceschi *et al.*, 1995).

Cases were 2569 women with incident, histologically-confirmed breast cancer (median age 55, range 23–74 years) admitted to major teaching and general hospitals of the study areas. Controls were 2588 women (median age 56, range 20–74 years) with no history of cancer admitted to the same hospitals for acute, non-neoplastic, nongynecological conditions, unrelated to hormonal or digestive tract diseases or to dietary-related conditions. Among controls, 22% were admitted for traumas, 33% for other orthopedic diseases, 15% for acute surgical conditions, 18% for eye diseases and 12% for other miscellaneous diseases. Less than 4% of cases and controls approached for interview refused to participate. The study was approved by the local ethics committees according to the rules at the time of data collection. All procedures were performed in accordance with the ethical standards laid down in the Declaration of Helsinki.

Cases and controls were interviewed in hospital by centrally trained interviewers, using a standard structured questionnaire. This included information on sociodemographic and anthropometric factors, lifestyle habits, including tobacco smoking, alcohol drinking and physical activity, as well as obstetric, gynecologic and general medical history, and family history of cancer. Subjects’ usual diet in the previous 2 years was assessed through a food frequency questionnaire (FFQ). Subjects were asked to indicate their average weekly consumption of 78 food items or food groups. Open questions were used to report foods/recipes eaten at least once a week not included in the FFQ list. The FFQ included also a few questions aiming at assessing fat intake pattern as well as information on salt and garlic use. Intakes lower than once a week, but at least once per month, were coded as 0.5/week. Nutrient and total energy intake were determined using an Italian food composition database (Salvini *et al*., 1998; Gnagnarella *et al.*, 2004).

We calculated the DRRD score according to Kang *et al.* (2020), on the basis of the following eight dietary components: cereal fiber, coffee (caffeinated and decaffeinated), total fruit, nuts, ratio of polyunsaturated to saturated fats, dietary GI, red and processed meat and sweetened beverages and fruit juices. We assigned a score between 1 and 5 according to quintile of consumption, in ascending order for cereal fiber, coffee, total fruit and ratio of polyunsaturated to saturated fats (i.e. factors associated to low diabetes risk), and in descending order for GI and red/processed meat (i.e. factors associated to high diabetes risk). Quintiles were derived among control women. The consumption of sweetened beverages and fruit juices was low in our population (i.e. 3094 (60%) women did not consume either sweetened beverages or fruit juices regularly); we, therefore, assigned a score of 5 to nonconsumers, a value of 3 to women drinking ≤2 drinks per week (i.e. the median value among control drinkers), and a value of 1 to women drinking more than two drinks per week. There was no specific question on nuts consumption in the FFQ; women reporting nuts consumption (*n* = 45) in the open questions of the FFQ were assigned a score of 2; a score of 1 was assigned to the remaining women. Due to the lack of trans fats information within the Italian food composition tables, trans fats intake could not be calculated and included in the score. For each woman, the DRRD score was obtained by summing up the scores in all the dietary components. The score thus theoretically ranged from 8 to 37, with higher values indicating greater adherence to the DRRD.

### Statistical analysis

Odds ratios (ORs) and the corresponding 95% confidence intervals (CIs) of breast cancer according to approximate quartiles (derived among controls) of the DRRD score and to a three-point increment in the score were estimated using unconditional logistic regression models. Two models were fitted: a first model included terms for study center, age and education; a second model included further terms for year of interview, BMI, physical activity, smoking, history of diabetes, parity, menopausal status and age at menopause, use of oral contraceptives and hormone replacement therapy, family history of breast cancer, alcohol intake and total energy intake. Trends in ORs across score quartiles were evaluated by including an ordinal variable for quartiles in the logistic regression models. A few missing data in the adjustment factors were replaced by the median value (continuous variables) or mode category (categorical variables) according to case/control status. We conducted the following sensitivity analyses: (1) we included in the models the adjustment for total vegetable intake and (2) for weight change since age 30 (the information was not available for 148 women), (3) excluded diabetic women from the analyses and (4) assessed the association between the DRRD score and breast cancer excluding each score component in turn from the DRRD score calculation. Subgroup analyses were performed according to menopausal status, education, parity, BMI and smoking status. Heterogeneity across strata was tested by a likelihood ratio test comparing models with and without interactions terms for the score quartile variables and the subgroup factors.

All the analyses were conducted using the SAS software, version 9.4 (SAS Institute, Inc., Cary, North Carolina, USA).

## Results

Table 1 gives the distribution of breast cancer cases and controls according to age and selected covariates. Cases were more educated than controls and have more frequently a family history of the disease; they also tended to have lower parity and to report more frequently a history of diabetes.

**Table 1:** Distribution of 2569 breast cancer cases and 2588 controls by age and other selected covariates (Italy, 1991–1994).

	Cases, *n* (%)	Controls, *n* (%)
Age group (years)		
<40	206 (8.0)	257 (9.9)
40-49	633 (24.6)	512 (19.8)
50-59	809 (31.5)	808 (31.2)
60-69	733 (38.5)	775 (30.0)
≥70	188 (7.3)	236 (9.1)
Education^a^ (years)		
<7	1259 (49.3)	1569 (61.2)
7-11	714 (28.0)	642 (25.0)
≥12	582 (22.8)	354 (13.8)
Parity^a^		
Nulliparae	401 (15.6)	380 (14.7)
1	584 (22.8)	494 (19.1)
2	968 (37.7)	909 (35.2)
3	406 (15.8)	489 (18.9)
≥4	207 (8.1)	314 (12.2)
Menopausal status^a^		
Premenopausal	988 (38.5)	843 (32.6)
Postmenopausal	1578 (61.5)	1745 (67.4)
History of diabetes		
No	2452 (95.5)	2489 (96.2)
Yes	117 (4.5)	99 (3.8)

a The sum does not add up to the total because of some missing values.

Table 2 provides the ORs and corresponding 95% CIs of breast cancer according to the DRRD score. The DRRD score was inversely related to the risk of breast cancer. ORs derived from models with minimal adjustment and those derived from models adjusted for several covariates were very similar. Based on the fully adjusted models, the OR for a three-point increment in the score was 0.93 (95% CI, 0.89–0.98), and women in the highest quartile of the DRRD score had a 24% reduced risk of breast cancer (95% CI, 11–36%) compared to those in the lowest quartile.

**Table 2:** Odds ratios (ORs) and corresponding 95% confidence intervals (CIs) for 2569 cases of breast cancer and 2588 controls, according to approximate quartiles of the diabetes risk reduction (DRRD) score (Italy, 1992–1994)

	Cases, *N* (%)	Controls, *N* (%)	OR^a^ (95% CI)	OR^b^ (95% CI)
DRRD score, quartiles				
I (≤20)	706 (27.5)	673 (26.0)	1^c^	1^c^
2 (21-22)	523 (20.4)	516 (19.9)	0.92 (0.78–1.09)	0.96 (0.82–1.14)
3 (23-25)	776 (30.2)	761 (29.4)	0.91 (0.79–1.06)	0.91 (0.78–1.06)
4 (≥26)	564 (22.0)	638 (24.7)	0.77 (0.65–0.90)	0.76 (0.64–0.89)
χ^2^ Trend (*P* value)			9.2 (0.002)	10.5 (0.001)
Three-point increment			0.94 (0.90–0.98)	0.93 (0.89–0.98)

a Adjusted for study center, age and education.

b Further adjusted for year of interview, BMI, physical activity, smoking, history of diabetes, parity, menopausal status and age at menopause, use of oral contraceptives and hormone replacement therapy, family history of breast cancer, alcohol intake and total energy intake.

c Reference category.

Further adjustment for total vegetable consumption (OR for a three-point increment in the score: 0.95, 95% CI, 0.90–0.996; OR_Q4vsQ1_: 0.80, 95% CI, 0.67–0.94) or weight change since age 30 (OR for a three-point increment: 0.93, 95% CI, 0.88–0.97; OR_Q4vsQ1_: 0.74, 95% CI, 0.62–0.87), as well as the exclusion of diabetic women from the analyses (OR for a three-point increment: 0.93, 95% CI, 0.89–0.98; OR_Q4vsQ1_: 0.74, 95% CI, 0.63–0.88) did not materially affect any of the results. Fairly consistent results were found after removing each dietary factor in turn from the DRRD score calculation; the ORs for the highest *versus* the lowest score quartile varied between 0.70 (with the exclusion of the cereal fiber component) and 0.85 (of borderline significance, with the exclusion of the dietary GI component or the polyunsaturated:saturated fats component) (Supplementary Information, Supplemental digital content 1, http://links.lww.com/EJCP/A330).

**Fig. 1:**
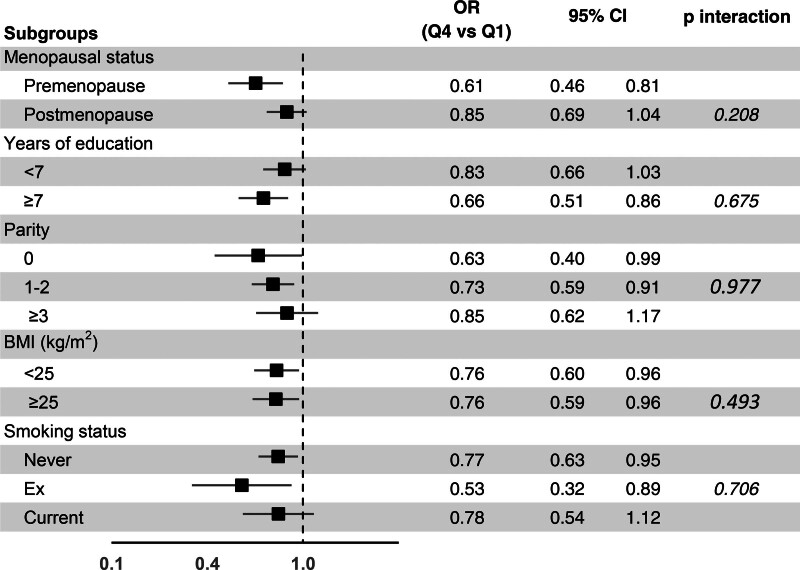
Odds ratios (ORs) of breast cancer for the highest (Q4) versus the lowest quartile (Q1) of the diabetes risk reduction diet (DRRD) score, with corresponding 95% confidence intervals (CIs), in selected subgroups (Italy, 1992–1994). The ORs were adjusted for study center, age, education, year of interview, BMI, physical activity, smoking, history of diabetes, parity, menopausal status and age at menopause, use of oral contraceptives and hormone replacement therapy, family history of breast cancer, alcohol intake and total energy intake, unless the covariate was the stratification factor. The lowest DRRD score quartile was the reference category in the analyses. Tests for interaction considered all the four quartiles of the DRRD score.

Figure 1 shows the results of the subgroup analysis. The inverse association between the DRRD score and breast cancer was observed in all the subgroups examined, being apparently stronger among premenopausal, more educated, nulliparous and ex-smoker women; however, tests for interaction did not reveal significant heterogeneity across all the strata considered.

## Discussion

In the present large multicentric study from Italy, a score measuring adherence to a DRRD and based on eight dietary components was significantly inversely related with the risk of breast cancer. After allowance for a number of potential confounders, including BMI and total energy intake, women in the highest score quartile had a 24% reduced risk of breast cancer compared to those in the lowest quartile. The inverse association between the DRRD score and the risk of breast cancer was observed in all the subgroups considered, although it was somehow more evident among premenopausal, more educated, nulliparous and ex-smoker women. In any case, tests for interaction did not detect any significant heterogeneity across strata.

The only other previous study investigating the association of the DRRD with the risk of breast cancer was a pooled analysis of two large US cohort studies (i.e. the Nurses’ Health Study NHS, and the NHSII), following 180 000 women for ≥26 years (Kang *et al.*, 2020). The study found a modestly lower breast cancer risk among women in the highest quintile of the DRRD score compared to those in the lowest quintile (multiple-adjusted hazard ratio, HR, 0.89, 95% CI, 0.84–0.95), which was slightly attenuated after adjusting for weight change since age 18 (HR, 0.92, 95% CI, 0.87–0.98). In our study, further adjustment for weight change since age 30 did not impact the results.

In the assessment of the relationship between diet and the risk of chronic diseases, the analysis of dietary patterns represents an alternative and complementary approach to the analysis of individual foods or nutrients (Hu, 2002). Dietary patterns, which capture the consumption of multiple dietary factors, may be more etiologically relevant than the traditional individual foods/nutrients analysis, particularly when only a few individual dietary factors have shown consistent associations with the disease, such as for breast cancer. The DRRD is a dietary pattern specifically developed for the reduction of diabetes risk. Subjects compliant to the DRRD have a high consumption of cereal fiber, coffee, fruit, nuts and a high ratio of polyunsaturated to saturated fats (i.e. factors inversely related to type II diabetes), and low GI, low intake of red/processed meat, sweetened beverages and fruit juices and trans fats (i.e. factors directly associated to type II diabetes). According to a recent meta-analysis of prospective studies, high intake of total fiber was weakly, but significantly, inversely associated with the risk of breast cancer; when investigating different sources of fiber, however, the meta-analysis did not find any association with the intake of cereal fiber (pooled RR for high *versus* low consumption 0.97, 95% CI, 0.93–1.01, based on 10 studies) (Farvid *et al.*, 2020). While some studies suggested a favorable role of high fruit consumption (Farvid *et al.*, 2019), other studies did not find any association (Emaus *et al.*, 2016), or reported inverse associations restricted to selected subtypes of breast cancer defined by hormone receptor status (Jung *et al.*, 2013). In any case, if anything, the association is likely modest (Aune *et al.*, 2012). Nuts consumption was associated with a marginally significant 10% reduced risk of breast cancer in a meta-analysis of six studies (Zhang *et al.*, 2020). Coffee may decrease the risk of postmenopausal breast cancer, but, again, the association is probably modest (Lafranconi *et al.*, 2018). High intakes of saturated and (n-3) polyunsaturated fats may, respectively, increase and decrease the risk of breast cancer, but the evidence is conflicting (Buja *et al.*, 2020). High GI or GL diets are not, or at most only weakly, directly associated with breast cancer (Turati *et al.*, 2019). Some studies indicated an increased risk of breast cancer for high consumption of red and processed meat (Inoue-Choi *et al.*, 2016; Diallo *et al.*, 2018); however, according to recent meta-analyses, only the intake of processed meat, but not of red meat, increases the risk of breast cancer (by 6–9% when consumed in higher amounts) (Anderson *et al.*, 2018; Farvid *et al.*, 2018). The few studies investigating sugary drinks in relation to breast cancer gave conflicting results (Makarem *et al.*, 2018; Chazelas *et al.*, 2019). We did not include the trans fats component in the DRRD score; however, the intake of trans fats does not appear to increase the risk of breast cancer (Anjom-Shoae *et al.*, 2020). Thus, the reduction in the risk of breast cancer for high adherence to the DRRD was evident even though no strong associations were observed for each of the nutritional components of the DRRD score. Biologic interactions may exist between the various dietary factors of the DRRD pattern. In addition, while the effects of individual dietary factors are examined against the background of average risk associated with other dietary exposures, the use of an inclusive dietary score can account for extremes of cumulative exposure, in the absence of other major nutritional effects (Jacques and Tucker, 2001).

Seeds oil is commonly consumed in the USA, where the score was derived, and is a major dietary contributor of polyunsaturated fats in the country. In the Mediterranean area, olive oil (in particular the extra virgin type) is the main source of dietary fat and is a major source of monounsaturated fatty acids (Trichopoulou *et al.*, 2014). Consumption of olive oil has been favorably related to several diseases, including cardiovascular diseases, various neoplasms (including breast cancer) (Pelucchi *et al.*, 2011) and diabetes (Schwingshackl *et al.*, 2017). When we used the ratio of monounsaturated+polyunsaturated to saturated fats (instead of that of polyunsaturated to saturated fats) in the score calculation, we observed similar results for the overall DRRD score (OR for a three-point increment in the score: 0.95, 95% CI, 0.90–0.99; OR for the highest *versus* the lowest score quartile: 0.80, 95% CI, 0.68–0.93).

Given the retrospective design of the study, potential selection and information bias should be considered. However, the exclusion from the control group of patients admitted for chronic and gynecologic conditions or diseases related to diet modifications or known risk factors for breast cancer, the very high participation rate (>95% for both cases and control), the similar catchment areas and interview setting for cases and controls and the lack of awareness in this population of a possible role of diet on breast cancer risk weigh against these biases. In addition, the FFQ was tested for validity and reproducibility with satisfactory results (Franceschi *et al.*, 1993; Decarli *et al.*, 1996). Although we were able to adjust for a number of potential confounding factors, including BMI and total energy intake, some residual confounding cannot be excluded. However, the fact that OR estimates did not change when several additional potential confounders were added to the models argues against major residual confounding. We could not include *trans* fats in the DRRD score, as proposed by Kang *et al.* (2020), as no information on the content of trans fats in Italian foods is available from food composition tables. Trans fats come primarily from industrial sources, by partial hydrogenation of edible oils. The major sources of trans fats are margarine, fried fast foods, and highly industrially processed foods, including packaged snacks and baked products. Compared to other western countries, consumption of highly industrially processed foods is lower in southern European countries, Italy included (Slimani *et al.*, 2009). As for margarine, in our study, only 2.5, 1.2 and 3.9% of women indicated it as the main fat source, respectively, for cooking meat, frying or seasoning pasta.

### Conclusion

Our study suggests that higher adherence to a dietary pattern for diabetes risk reduction lowers the risk of breast cancer. It remains unclear whether this is due to a direct effect of such diet on glycemia and related factors (e.g. IGF-1) or to other mechanisms related to the individual components and their combination. The observation that the effect is greater in premenopausal women would at least in part support the latter hypothesis.

## Acknowledgements

This work was supported by the AIRC (Associazione Italiana per la Ricerca sul Cancro) Foundation, the Italian League for the Fight against Cancer and Department funding.

### Conflicts of interest

There are no conflicts of interest.

## Supplementary Material

## References

[R1] Anderson JJ Darwis NDM Mackay DF Celis-Morales CA Lyall DM Sattar N. (2018). Red and processed meat consumption and breast cancer: UK Biobank cohort study and meta-analysis. Eur J Cancer 90:73–82. 29274927 10.1016/j.ejca.2017.11.022

[R2] Anjom-Shoae J Sadeghi O Larijani B Esmaillzadeh A (2020). Dietary intake and serum levels of trans fatty acids and risk of breast cancer: a systematic review and dose-response meta-analysis of prospective studies. Clin Nutr 39:755–764. 30954361 10.1016/j.clnu.2019.03.024

[R3] Aune D Chan DS Vieira AR Rosenblatt DA Vieira R Greenwood DC Norat T (2012). Fruits, vegetables and breast cancer risk: a systematic review and meta-analysis of prospective studies. Breast Cancer Res Treat 134:479–493. 22706630 10.1007/s10549-012-2118-1

[R4] Bagnardi V Rota M Botteri E Tramacere I Islami F Fedirko V. (2015). Alcohol consumption and site-specific cancer risk: a comprehensive dose-response meta-analysis. Br J Cancer 112:580–593. 25422909 10.1038/bjc.2014.579 PMC4453639

[R6] Boyle P Boniol M Koechlin A Robertson C Valentini F Coppens K. (2012). Diabetes and breast cancer risk: a meta-analysis. Br J Cancer 107:1608–1617. 22996614 10.1038/bjc.2012.414 PMC3493760

[R7] Buckland G Travier N Cottet V González CA Luján-Barroso L Agudo A. (2013). Adherence to the mediterranean diet and risk of breast cancer in the European prospective investigation into cancer and nutrition cohort study. Int J Cancer 132:2918–2927. 23180513 10.1002/ijc.27958

[R8] Buja A Pierbon M Lago L Grotto G Baldo V (2020). Breast cancer primary prevention and diet: an umbrella review. Int J Environ Res Public Health 17:E4731. 10.3390/ijerph17134731 PMC7369836 32630215

[R9] Carey VJ Walters EE Colditz GA Solomon CG Willett WC Rosner BA. (1997). Body fat distribution and risk of non-insulin-dependent diabetes mellitus in women. The Nurses’ Health Study. Am J Epidemiol 145:614–619. 9098178 10.1093/oxfordjournals.aje.a009158

[R10] Chan DSM Abar L Cariolou M Nanu N Greenwood DC Bandera EV. (2019). World Cancer Research Fund International: Continuous Update Project-systematic literature review and meta-analysis of observational cohort studies on physical activity, sedentary behavior, adiposity, and weight change and breast cancer risk. Cancer Causes Control 30:1183–1200. 31471762 10.1007/s10552-019-01223-w

[R11] Chazelas E Srour B Desmetz E Kesse-Guyot E Julia C Deschamps V. (2019). Sugary drink consumption and risk of cancer: results from NutriNet-Santé prospective cohort. BMJ 366:l2408. 31292122 10.1136/bmj.l2408 PMC6614796

[R12] Decarli A Franceschi S Ferraroni M Gnagnarella P Parpinel MT La Vecchia C. (1996). Validation of a food-frequency questionnaire to assess dietary intakes in cancer studies in Italy. Results for specific nutrients. Ann Epidemiol 6:110–118. 8775590 10.1016/1047-2797(95)00129-8

[R13] Diallo A Deschasaux M Latino-Martel P Hercberg S Galan P Fassier P. (2018). Red and processed meat intake and cancer risk: results from the prospective NutriNet-Santé cohort study. Int J Cancer 142:230–237. 28913916 10.1002/ijc.31046

[R14] Eliassen AH Hendrickson SJ Brinton LA Buring JE Campos H Dai Q. (2012). Circulating carotenoids and risk of breast cancer: pooled analysis of eight prospective studies. J Natl Cancer Inst 104:1905–1916. 23221879 10.1093/jnci/djs461 PMC3525817

[R15] Emaus MJ Peeters PH Bakker MF Overvad K Tjønneland A Olsen A. (2016). Vegetable and fruit consumption and the risk of hormone receptor-defined breast cancer in the EPIC cohort. Am J Clin Nutr 103:168–177. 26607934 10.3945/ajcn.114.101436

[R16] Farvid MS Chen WY Michels KB Cho E Willett WC Eliassen AH (2016). Fruit and vegetable consumption in adolescence and early adulthood and risk of breast cancer: population based cohort study. BMJ 353:i2343. 27170029 10.1136/bmj.i2343 PMC5068921

[R17] Farvid MS Chen WY Rosner BA Tamimi RM Willett WC Eliassen AH (2019). Fruit and vegetable consumption and breast cancer incidence: repeated measures over 30 years of follow-up. Int J Cancer 144:1496–1510. 29978479 10.1002/ijc.31653 PMC6440478

[R18] Farvid MS Spence ND Holmes MD Barnett JB (2020). Fiber consumption and breast cancer incidence: a systematic review and meta-analysis of prospective studies. Cancer 126:3061–3075. 32249416 10.1002/cncr.32816

[R19] Farvid MS Stern MC Norat T Sasazuki S Vineis P Weijenberg MP. (2018). Consumption of red and processed meat and breast cancer incidence: a systematic review and meta-analysis of prospective studies. Int J Cancer 143:2787–2799. 30183083 10.1002/ijc.31848 PMC8985652

[R20] Franceschi S Favero A La Vecchia C Negri E Dal Maso L Salvini S. (1995). Influence of food groups and food diversity on breast cancer risk in Italy. Int J Cancer 63:785–789. 8847134 10.1002/ijc.2910630606

[R21] Franceschi S Negri E Salvini S Decarli A Ferraroni M Filiberti R. (1993). Reproducibility of an Italian food frequency questionnaire for cancer studies: results for specific food items. Eur J Cancer 29A:2298–2305. 8110502 10.1016/0959-8049(93)90225-5

[R22] Gallagher EJ LeRoith D (2010). The proliferating role of insulin and insulin-like growth factors in cancer. Trends Endocrinol Metab 21:610–618. 20663687 10.1016/j.tem.2010.06.007 PMC2949481

[R23] Giovannucci E Harlan DM Archer MC Bergenstal RM Gapstur SM Habel LA. (2010). Diabetes and cancer: a consensus report. CA Cancer J Clin 60:207–221. 20554718 10.3322/caac.20078

[R24] Gnagnarella P Parpinel M Salvini S Franceschi S Palli D Boyle P (2004). The update of the Italian food composition database. J Food Comp Analysis 17:509–522.

[R25] Hernandez AV Guarnizo M Miranda Y Pasupuleti V Deshpande A Paico S. (2014). Association between insulin resistance and breast carcinoma: a systematic review and meta-analysis. PLoS One 9:e99317. 24911052 10.1371/journal.pone.0099317 PMC4049776

[R26] Hu FB (2002). Dietary pattern analysis: a new direction in nutritional epidemiology. Curr Opin Lipidol 13:3–9. 11790957 10.1097/00041433-200202000-00002

[R27] Inoue-Choi M Sinha R Gierach GL Ward MH (2016). Red and processed meat, nitrite, and heme iron intakes and postmenopausal breast cancer risk in the NIH-AARP Diet and Health Study. Int J Cancer 138:1609–1618. 26505173 10.1002/ijc.29901 PMC4724256

[R28] Jacques PF Tucker KL (2001). Are dietary patterns useful for understanding the role of diet in chronic disease? Am J Clin Nutr 73:1–2. 11124739 10.1093/ajcn/73.1.1

[R29] Jung S Spiegelman D Baglietto L Bernstein L Boggs DA van den Brandt PA. (2013). Fruit and vegetable intake and risk of breast cancer by hormone receptor status. J Natl Cancer Inst 105:219–236. 23349252 10.1093/jnci/djs635 PMC3593764

[R30] Kang JH Peng C Rhee JJ Farvid MS Willett WC Hu FB. (2020). Prospective study of a diabetes risk reduction diet and the risk of breast cancer. Am J Clin Nutr 112:1492–1503. 33022701 10.1093/ajcn/nqaa268 PMC7727476

[R31] Key T Appleby P Barnes I Reeves G; Endogenous Hormones and Breast Cancer Collaborative Group. (2002). Endogenous sex hormones and breast cancer in postmenopausal women: reanalysis of nine prospective studies. J Natl Cancer Inst 94:606–616. 11959894 10.1093/jnci/94.8.606

[R32] Key TJ Appleby PN Reeves GK Roddam AW; Endogenous Hormones and Breast Cancer Collaborative Group. (2010). Insulin-like growth factor 1 (IGF1), IGF binding protein 3 (IGFBP3), and breast cancer risk: pooled individual data analysis of 17 prospective studies. Lancet Oncol 11:530–542. 20472501 10.1016/S1470-2045(10)70095-4 PMC3113287

[R33] La Vecchia C Giordano SH Hortobagyi GN Chabner B (2011). Overweight, obesity, diabetes, and risk of breast cancer: interlocking pieces of the puzzle. Oncologist 16:726–729. 21632448 10.1634/theoncologist.2011-0050 PMC3228228

[R34] Lafranconi A Micek A De Paoli P Bimonte S Rossi P Quagliariello V Berretta M (2018). Coffee intake decreases risk of postmenopausal breast cancer: a dose-response meta-analysis on prospective cohort studies. Nutrients 10:E112. 10.3390/nu10020112 PMC5852688 29360766

[R35] Makarem N Bandera EV Lin Y Jacques PF Hayes RB Parekh N (2018). Consumption of sugars, sugary foods, and sugary beverages in relation to adiposity-related cancer risk in the Framingham offspring cohort (1991-2013). Cancer Prev Res (Phila) 11:347–358. 29674390 10.1158/1940-6207.CAPR-17-0218 PMC7225083

[R36] Pelucchi C Bosetti C Negri E Lipworth L La Vecchia C (2011). Olive oil and cancer risk: an update of epidemiological findings through 2010. Curr Pharm Des 17:805–812. 21443483 10.2174/138161211795428920

[R37] Rhee JJ Mattei J Hughes MD Hu FB Willett WC (2015). Dietary diabetes risk reduction score, race and ethnicity, and risk of type 2 diabetes in women. Diabetes Care 38:596–603. 25592193 10.2337/dc14-1986 PMC4370327

[R38] Salvini S Parpinel M Gnagnarella P Maisonneuve P Turrini A (1998). Banca di composizione degli alimenti per studi epidemiologici in Italia. Milano. Istituto Europeo di Oncologia.

[R39] Schernhammer ES Holly JM Hunter DJ Pollak MN Hankinson SE (2006). Insulin-like growth factor-I, its binding proteins (IGFBP-1 and IGFBP-3), and growth hormone and breast cancer risk in The Nurses Health Study II. Endocr Relat Cancer 13:583–592. 16728584 10.1677/erc.1.01149

[R40] Schwingshackl L Lampousi AM Portillo MP Romaguera D Hoffmann G Boeing H (2017). Olive oil in the prevention and management of type 2 diabetes mellitus: a systematic review and meta-analysis of cohort studies and intervention trials. Nutr Diabetes 7:e262. 28394365 10.1038/nutd.2017.12 PMC5436092

[R41] Slimani N Deharveng G Southgate DA Biessy C Chajès V van Bakel MM. (2009). Contribution of highly industrially processed foods to the nutrient intakes and patterns of middle-aged populations in the European Prospective Investigation into Cancer and Nutrition study. Eur J Clin Nutr 63 (Suppl 4):S206–S225. 19888275 10.1038/ejcn.2009.82

[R42] Trichopoulou A Martínez-González MA Tong TY Forouhi NG Khandelwal S Prabhakaran D. (2014). Definitions and potential health benefits of the Mediterranean diet: views from experts around the world. BMC Med 12:112. 25055810 10.1186/1741-7015-12-112 PMC4222885

[R43] Turati F Carioli G Bravi F Ferraroni M Serraino D Montella M. (2018). Mediterranean diet and breast cancer risk. Nutrients 10:E326. 10.3390/nu10030326 PMC5872744 29518016

[R44] Turati F Dalmartello M Bravi F Serraino D Augustin L Giacosa A. (2020). Adherence to the world cancer research fund/American institute for cancer research recommendations and the risk of breast cancer. Nutrients 12:E607. 10.3390/nu12030607 PMC7146587 32110887

[R45] Turati F Galeone C Augustin LSA La Vecchia C (2019). Glycemic index, glycemic load and cancer risk: an updated meta-analysis. Nutrients 11:E2342. 10.3390/nu11102342 PMC6835610 31581675

[R46] van den Brandt PA Ziegler RG Wang M Hou T Li R Adami HO. (2021). Body size and weight change over adulthood and risk of breast cancer by menopausal and hormone receptor status: a pooled analysis of 20 prospective cohort studies. Eur J Epidemiol 36:37–55. 33128203 10.1007/s10654-020-00688-3 PMC7847460

[R47] Wolf I Sadetzki S Catane R Karasik A Kaufman B (2005). Diabetes mellitus and breast cancer. Lancet Oncol 6:103–111. 15683819 10.1016/S1470-2045(05)01736-5

[R48] Xiao Y Xia J Li L Ke Y Cheng J Xie Y. (2019). Associations between dietary patterns and the risk of breast cancer: a systematic review and meta-analysis of observational studies. Breast Cancer Res 21:16. 30696460 10.1186/s13058-019-1096-1 PMC6352362

[R49] Zhang D Dai C Zhou L Li Y Liu K Deng YJ. (2020). Meta-analysis of the association between nut consumption and the risks of cancer incidence and cancer-specific mortality. Aging (Albany NY) 12:10772–10794. 32487780 10.18632/aging.103292 PMC7346045

