# Supplementary material for: Diabetes risk reduction diet and the risk of breast cancer
Source: Eur J Cancer Prev. 2021 Aug 16;31(4):339–45. doi: 10.1097/CEJ.0000000000000709 (PMC9889193; doi:10.1097/CEJ.0000000000000709)
Supplement: Supplementary file 1 [file ejcp-31-339-s001.pdf]

Supplementary Figure 1. Odds ratios[a] (OR) of breast cancer and corresponding 95% confidence interval (CI) for the highest (Q4) *versus* the lowest quartile (Q1) of the diabetes risk reduction diet (DRRD) score after excluding from the calculation each of its components (Italy, 1992–1994).

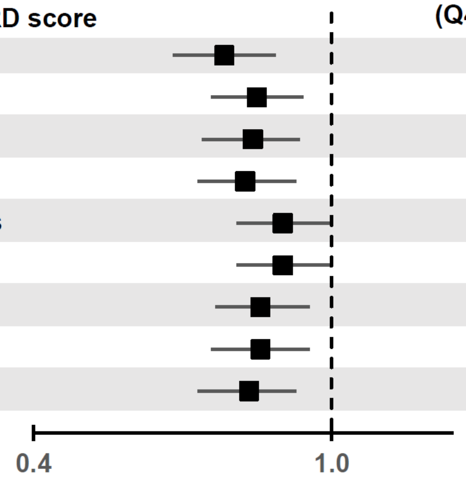

Factor excluded from the DRRD score	OR (Q4 vs Q1)	95% CI	
Cereal fiber	0.70	0.59	0.83
Coffee	0.78	0.67	0.91
Fruit	0.77	0.65	0.90
Nuts	0.75	0.64	0.89
Polyunsaturated:saturated fats	0.85	0.73	1.00
Glycemic index	0.85	0.73	1.00
Red and processed meat	0.79	0.68	0.93
Soft drinks and fruit juices	0.79	0.67	0.93
**Original DRRD score**	0.76	0.64	0.89

[a]Adjusted for study center, age, education, year of interview, body mass index, physical activity, smoking, history of diabetes, parity, menopausal status and age at menopause, use of oral contraceptives and hormone replacement therapy, family history of breast cancer, alcohol intake and total energy intake. The lowest DRRD score quartile was the reference category in the analyses.
